# Association of Post-operative Systolic Blood Pressure Variability With Mortality After Coronary Artery Bypass Grafting

**DOI:** 10.3389/fcvm.2021.717073

**Published:** 2021-08-12

**Authors:** Zhuoming Zhou, Jiantao Chen, Guangguo Fu, Xiaodong Zhuang, Jian Hou, Sida Chen, Suiqing Huang, Yuan Yue, Liqun Shang, Keke Wang, Linhua Lv, Mengya Liang, Zhongkai Wu

**Affiliations:** ^1^Department of Cardiac Surgery, The First Affiliated Hospital of Sun Yat-Sen University, Guangzhou, China; ^2^NHC Key Laboratory of Assisted Circulation, Sun Yat-Sen University, Guangzhou, China; ^3^Department of Cardiology, The First Affiliated Hospital of Sun Yat-Sen University, Guangzhou, China; ^4^Center for Information Technology & Statistics, The First Affiliated Hospital of Sun Yat-Sen University, Guangzhou, China; ^5^Department of Emergency, The First Affiliated Hospital of Sun Yat-Sen University, Guangzhou, China

**Keywords:** coronary artery bypass grafting, blood pressure, variability, mortality, intensive care

## Abstract

**Background:** Blood pressure variability (BPV) has long been considered a risk factor for cardiovascular events. We aimed to investigate whether post-operative systolic BPV was associated with early and late all-cause mortality in patients undergoing coronary artery bypass grafting (CABG).

**Methods:** Clinical variables and blood pressure records within the first 24 h in the post-operative intensive care unit stay from 4,509 patients operated on between 2001 and 2012 were extracted from the Medical Information Mart for Intensive Care III (MIMIC-III) database. BPV was measured as the coefficient of the variability of systolic blood pressure, and we compared patients in the highest quartile with patients in the other three quartiles.

**Results:** After full adjustment, patients in the highest quartile of BPV were at a higher risk of intensive care unit mortality (OR = 2.02, 95% CI: 1.11–3.69), 30-day mortality (OR = 1.92, 95% CI: 1.22–3.02), and 90-day mortality (HR = 1.64, 95% CI: 1.19–2.27). For 2,892 patients with a 4-year follow-up, the association between a higher post-operative BPV and the risk of 4-year mortality was not significant (HR = 1.17, 95% CI: 0.96–1.42). The results were supported by the comparison of survival curves and remained generally consistent in the subgroup analyses and sensitivity analyses.

**Conclusions:** Our findings demonstrated that in patients undergoing CABG, a higher post-operative BPV was associated with a higher risk of early mortality while the association was not significant for late mortality. Post-operative BPV can support doctors in identifying patients with potential hemodynamic instability and making timely clinical decisions.

## Introduction

Elevated blood pressure (BP) has been demonstrated to be a dominant contributor and risk factor for a poor cardiovascular prognosis (1–3). However, BP is a dynamic physiological parameter, and the average of multiple BP readings over a period of time is insufficient to reflect the real risks of patients (4). A growing number of studies have indicated that blood pressure variability (BPV) over hours, days, and years is associated with cardiovascular events, including heart failure (5), atrial fibrillation (6), coronary artery disease, stroke, and both cardiovascular and all-cause mortality in different non-surgical populations (7–9), independent of the corresponding mean BP. The underlying mechanisms remain incompletely understood, but arterial compliance and endothelial dysfunction may be involved (10).

Coronary artery bypass grafting (CABG) is a surgical treatment for patients with severe coronary artery disease, and many of them have concomitant diseases such as hypertension, diabetes mellitus, and congestive heart failure (CHF) (11). Therefore, fluctuations of BP due to cardiac insufficiency, unsatisfactory vascular quality, and inappropriate BP management frequently occur pre- and post-operatively. Previous publications have shown that preoperative long-term BPV and intraoperative oscillation of BP can predict short-term outcomes in patients undergoing CABG (12, 13). However, it is difficult to regularly measure and control preoperative long-term BP, while post-operative BP can be routinely monitored and adjusted in the intensive care unit (ICU) after surgery.

As systolic blood pressure (SBP) has been considered to have more clinical significance than diastolic blood pressure (DBP) (14, 15), the aim of our study was to further explore the association between post-operative systolic BPV and early and late all-cause mortality in patients undergoing CABG and discuss its prognostic and therapeutic implications for optimizing post-operative BP management.

## Methods

### Data Source and Study Population

Data were obtained from the Medical Information Mart for Intensive Care III (MIMIC-III) database, which is a large, freely available database with information from more than 40,000 patients who had critical care unit stays at the Beth Israel Deaconess Medical Center between 2001 and 2012 (16). Our right to access the database and acquire the data was approved by the institutional review board of the Massachusetts Institute of Technology (Cambridge, MA, USA) after one of our authors (Zhou) finished the online training for the Collaborative Institutional Training Initiative program of the National Institutes of Health (Record ID 35971811).

Among all of the patients in the MIMIC-III database, 5,007 consecutive patients undergoing CABG between 2001 and 2012 were included. The exclusion criteria were as follows: (1) missing BP measurement records (*n* = 274); (2) death within the first 24 h of post-operative ICU admission (*n* = 6); (3) length of ICU stay <24 h (*n* = 188); (4) BP measured <10 times within the first 24 h of post-operative ICU admission (*n* = 28); and (5) Patients with abnormal times (more than 1,000 times) of BP measurement (*n* = 2). Finally, a total of 4,509 patients were included in the study population.

### Data Extraction

All data were obtained and extracted using the Structured Query Language (SQL), and pgAdmin4 for Post-greSQL was used as the administrative platform. The extracted data included: (1) demographics: age, sex, and ethnicity; (2) comorbidities defined using the International Classification of Diseases, Ninth Revision, Clinical Modification (ICD-9-CM) codes, including CHF, hypertension, acute myocardial infarction (AMI), diabetes mellitus, respiratory failure, peripheral vascular disease, and end stage renal disease (ESRD); (3) concomitant surgical procedures: cardiopulmonary bypass, number of revascularized arteries, single internal thoracic artery (SITA) grafting, and concomitant valvular surgery; (4) intensive care scores: Sequential Organ Failure Assessment (SOFA) and Simplified Acute Physiology Score II (SAPS II); (5) vital signs: SBP, DBP, heart rate, and percutaneous oxygen saturation (SpO_2_); (6) antihypertensive medication including angiotensin-converting enzyme inhibitors (ACEIs), angiotensin receptor blockers (ARBs), calcium channel blockers (CCBs), beta-blockers, and diuretics; and (7) vasoactive medication including dobutamine, dopamine, epinephrine, norepinephrine, phenylephrine, and vasopressin.

### BP Measurement and the Definition of BPV

Post-operative BP was continuously measured and recorded with a bedside BP monitor (Component Monitoring System IntelliVue MP-70; Philips Healthcare, Andover, MA) via an invasive arterial line within the first 24 h in the ICU. BP data was extracted from the database, and systolic and diastolic BPV was calculated and evaluated using the following parameters: (1) the coefficient of variation (CV) of BP, defined as the standardized deviation (SD) of SBP or DBP divided by the mean and expressed as a percentage; and (2) the SD of BP, defined as the SD of all SBP or DBP records for a patient within the first 24 h of ICU admission. Patients in the highest quartile group were defined as patients with higher BPV, and were compared with patients from the three lowest quartiles of variability.

### Definition of Outcomes and Follow-Up

All patients were followed up for at least 90 days, and the primary outcome was all-cause mortality occurring within 30 days after the CABG operation. ICU and 90-day mortality were considered secondary outcomes. For the 2,892 consecutive patients who underwent surgery between 2001 and 2008, they were followed up for at least 4 years; thus, 4-year all-cause mortality was set as an additional outcome for those patients.

### Statistical Analysis

The study population was categorized into two groups according to the highest and the other three CV quartiles of SBP. Continuous variables were presented as the mean ± SD and were compared by Student *t*-test. Categorical data were presented as numbers with proportions and were analyzed by χ^2^-tests. Logistic regression models and Cox proportional hazards models were applied for the univariable and multivariable analyses to identify the association between the highest BPV quartile and mortality. Model 1 was adjusted for key demographic characteristics like age and sex; Model 2 was adjusted for age, sex, and comorbidities including CHF, hypertension, AMI, diabetes mellitus, respiratory failure, peripheral vascular disease, and ESRD. To account for the post-operative antihypertensive and vasoactive medication within the first 24 h, they were adjusted in Model 3. Model 4 was adjusted for the variables in Model 3 plus the number of BP measurements within the first 24 h. The results were presented as odds ratios (ORs) or hazard ratios (HRs) and 95% confidence intervals (CIs). Survival curves at 30 days, 90 days, and 4 years were estimated using the Kaplan-Meier method and compared by the log-rank test. Subgroup analyses were performed with a logistic regression model in Model 4, according to age strata (<70 and ≥70 years), sex, CHF, hypertension, AMI, diabetes mellitus, and antihypertensive and vasoactive medication. The *P* for interaction was derived from a multivariable logistic regression model. Sensitivity analyses were performed by measuring BPV as SD quartiles of SBP, CV quartiles of DBP, and SD quartiles of DBP. Then, patients who were discharged or died within the first 12 h were excluded, and analyses of BPV within the first 12 h based on the remaining 4,670 patients were performed. Finally, we included patients who were discharged within 24 h or had BP measured <10 times to assess the robustness of our analysis. All tests were two-sided, and *P* < 0.05 were considered significant. All statistical analyses were performed using STATA, version 14.0 (StataCorp, College Station, TX).

## Results

### Characteristics of the Patients

In total, 4,509 patients who met the selection criteria were enrolled in our study, all of whom were followed for 90 days, and among these, 2,892 patients were followed for 4 years. The baseline characteristics of the patients grouped by CV quartiles of SBP are briefly summarized in Table 1. Compared with the other three quartiles, patients in the highest BPV group were older, tend to be female, and were more likely to have CHF, diabetes mellitus and ESRD, higher BP, more BP measurements, higher intensive care scores, and less antihypertensive medication usage. The ICD-9-CM codes of the included comorbidities were summarized in Supplementary Table 1.

**Table 1:** Baseline characteristics of patients undergoing CABG, categorized by the coefficient of variation quartiles of post-operative SBP.

**Variables**	**Coefficient of variation of SBP**		***P*-value**
	**Q1–3 (*n* = 3,382)**	**Q4 (*n* = 1,127)**	
**Demographics**			
Age, years	67.46 (10.89)	70.02 (10.36)	<0.001
Sex, female, *n* (%)	826 (24.4)	350 (31.1)	<0.001
**Ethnicity**, ***n*****(%)**			
White	2,377 (70.3)	793 (70.4)	0.256
Black	82 (2.4)	39 (3.5)	
Asian	68 (2.0)	19 (1.7)	
Others	855 (25.3)	276 (24.5)	
CV of SBP	11.59 (2.14)	18.31 (3.15)	<0.001
**Comorbidities**, ***n*****(%)**			
Congestive heart failure	874 (25.8)	350 (31.1)	0.001
Hypertension	2,305 (68.2)	773 (68.6)	0.786
Acute myocardial infarction	267 (7.9)	104 (9.2)	0.158
Diabetes mellitus	1,304 (38.6)	487 (43.2)	0.006
Respiratory failure	136 (4.0)	65 (5.8)	0.014
Peripheral vascular disease	185 (5.5)	92 (8.2)	0.001
End-stage renal disease	121 (3.6)	67 (5.9)	0.001
**Concomitant surgical procedures**			
Concomitant valvular surgery, *n* (%)	690 (20.4)	251 (22.3)	0.181
Cardiopulmonary bypass, *n* (%)	3,195 (94.5)	1,045 (92.7)	0.032
SITA grafting, *n* (%)	3,003 (88.8)	969 (86.0)	0.012
Number of revascularized arteries	2.24 (0.92)	2.20 (0.88)	0.195
**Scores**			
SOFA	4.76 (2.48)	5.37 (2.66)	<0.001
SAPSII	35.10 (11.31)	39.61 (12.39)	<0.001
**Vital signs**			
Number of BP measurement, times	37.15 (11.45)	42.65 (14.40)	<0.001
Mean SBP, mmHg	112.35 (9.72)	113.12 (9.62)	0.020
Mean DBP, mmHg	56.90 (7.60)	56.54 (7.02)	0.159
Heart rate, beats/minute	85.28 (9.83)	85.46 (9.42)	0.593
SpO_2_, %	98.02 (1.23)	98.05 (1.72)	0.532
**Medication**			
**Antihypertensive**, ***n*****(%)**	2,599 (76.8)	784 (69.6)	<0.001
ACEI	141 (4.2)	33 (2.9)	0.061
ARB	23 (0.7)	10 (0.9)	0.480
Beta-blocker	2,013 (59.5)	534 (47.4)	<0.001
CCB	135 (4.0)	34 (3.0)	0.136
Diuretics	2,331 (68.9)	714 (63.4)	0.001
**Vasoactive**, ***n*****(%)**	2,164 (64.0)	723 (64.2)	0.920
Dobutamine	68 (2.0)	31 (2.8)	0.142
Dopamine	71 (2.1)	34 (3.0)	0.077
Epinephrine	439 (13.0)	220 (19.5)	<0.001
Norepinephrine	275 (8.1)	128 (11.4)	0.001
Phenylephrine	2,089 (61.8)	691 (61.3)	0.786
Vasopressin	135 (4.0)	72 (6.4)	0.001

*The highest quartile (Q4) was compared with Q1–3. Continuous variables are presented as the mean (SD), and categorical variables are presented as numbers (%)*.

*ACEI, angiotensin-converting enzyme inhibitor; ARB, angiotensin receptor blocker; CABG, coronary artery bypass grafting; CCB, calcium channel blocker; CV, coefficient of variation; DBP, diastolic blood pressure; Q, quartile; SAPS II, Simplified Acute Physiology Score II; SBP, systolic blood pressure; SITA, single internal thoracic artery; SOFA, Sequential Organ Failure Assessment; SpO_2_, percutaneous oxygen saturation*.

### Systolic BPV and the Risk of ICU and 30-day Mortality

Higher ICU mortality and 30-day mortality in the highest CV quartile of SBP were found in the unadjusted and adjusted models (Models 1–4). After adjusting for age, sex, comorbidities, medications, and the number of BP measurements (Model 4), patients in the highest BPV group were still at higher risk of ICU mortality (Q_4_ vs. Q_1−3_: OR = 2.02, 95% CI: 1.11–3.69) and 30-day mortality (Q_4_ vs. Q_1−3_: OR = 1.92, 95% CI: 1.22–3.02) (Table 2).

**Table 2:** Association between the highest quartile (Q4) vs. Q1–3 for the coefficient of variation of SBP within the first 24 h of post-operative ICU admission and mortality.

**Events**	**No. of events (%)**	**Univariable analysis**	**Multivariable analysis**			
			**Model 1**	**Model 2**	**Model 3**	**Model 4**
		**OR (95% CI)**				
**ICU mortality**						
Q1–3	27 (0.80)	1.00 (Reference)				
Q4	23 (2.04)	2.59 (1.48–4.53)	2.40 (1.36–4.22)	2.19 (1.21–3.94)	2.17 (1.20–3.94)	2.02 (1.11–3.69)
**30-day mortality**						
Q1–3	49 (1.45)	1.00 (Reference)				
Q4	39 (3.46)	2.44 (1.59–3.73)	2.28 (1.48–3.5)	2.11 (1.35–3.28)	2.07 (1.33–3.23)	1.92 (1.22–3.02)
		**HR (95% CI)**				
**90-day mortality**						
Q1–3	96 (2.84)	1.00 (Reference)				
Q4	64 (5.68)	2.04 (1.48–2.79)	1.81 (1.32–2.49)	1.70 (1.24–2.34)	1.66 (1.21–2.29)	1.64 (1.19–2.27)
**4-year mortality**						
Q1–3	313 (15.03)	1.00 (Reference)				
Q4	159 (19.63)	1.32 (1.09–1.61)	1.19 (0.98–1.45)	1.17 (0.96–1.41)	1.16 (0.96–1.41)	1.17 (0.96–1.42)

*Model 1 was adjusted for age and sex*.

*Model 2 was adjusted for age, sex, congestive heart failure, hypertension, acute myocardial infarction, diabetes mellitus, respiratory failure, peripheral vascular disease, and end-stage renal disease*.

*Model 3 was adjusted for the variables in Model 2 plus post-operative antihypertensive and vasoactive medication within the first 24 h*.

*Model 4 was adjusted for the variables in Model 3 plus the number of blood pressure measurements within the first 24 h*.

*ICU, intensive care unit; Q, quartile; SBP, systolic blood pressure*.

### Systolic BPV and the Risk of 90-day and 4-year Mortality

The highest quartile of systolic BPV was significantly associated with a higher risk of 90-day morality in all models. The correlation remained unchanged after fully adjusting for the variables in Model 4 (Q_4_ vs. Q_1−3_: HR = 1.64, 95% CI: 1.19–2.27) (Table 2).

In the 2,892 consecutive patients who were followed up for 4 years, the univariate analysis indicated that patients with the highest systolic BPV were at higher risk of 4-year mortality. However, after fully adjusting for variables in the multivariable analyses, this association became statistically insignificant, although the trend still favored a higher risk in Q4 (Q_4_ vs. Q_1−3_: HR = 1.17, 95% CI: 0.96–1.42) (Table 2).

**Figure 1:**
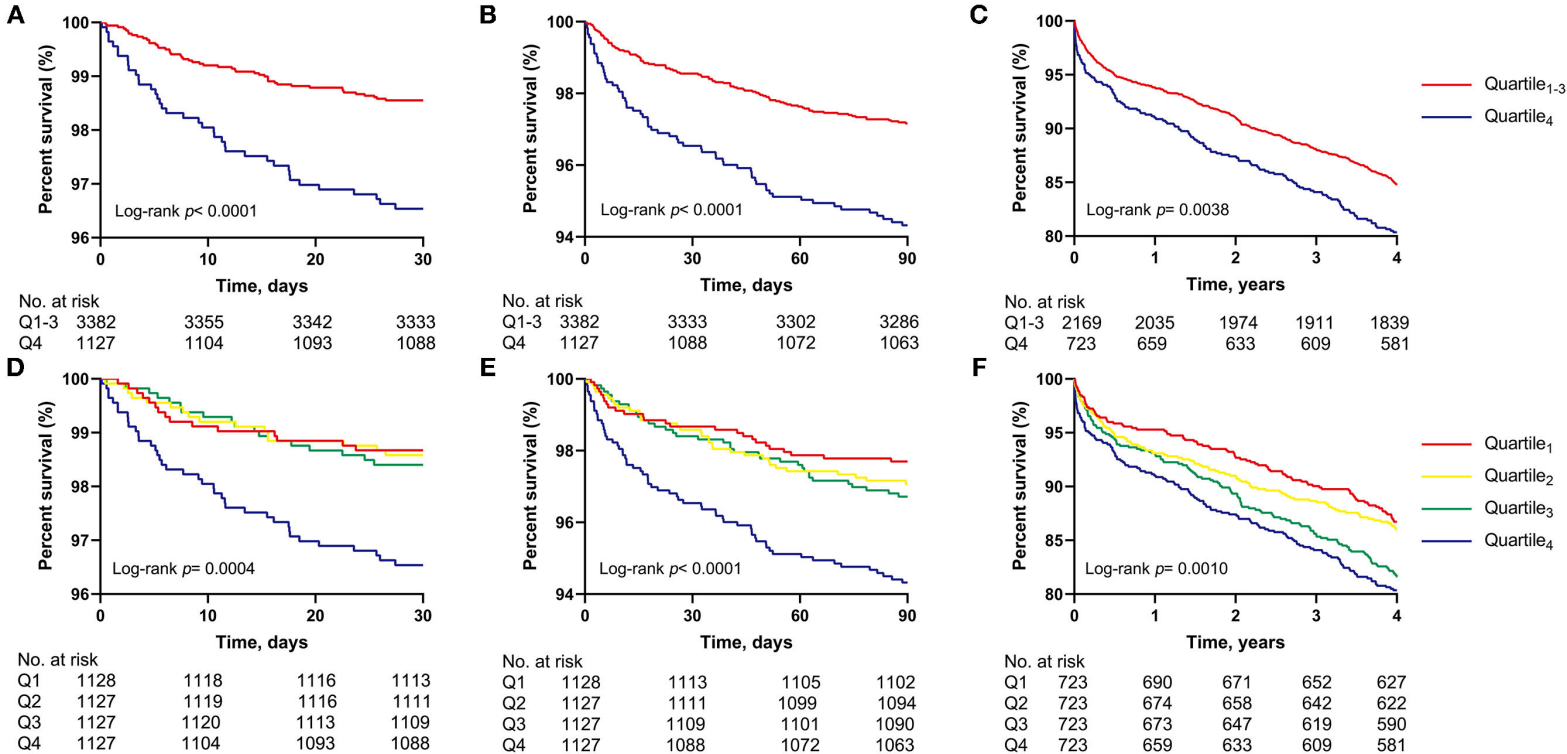
Kaplan-Meier survival analysis among patients stratified by quartiles of the coefficient of variation of post-operative systolic BPV. Comparison of **(A)** 30-day, **(B)** 90-day, and **(C)** 4-year survival of the highest quartile (Q4) vs. the other three quartiles (Q1–3); Comparison of **(D)** 30-day, **(E)** 90-day, and **(F)** 4-year survival of the four quartiles; BPV, blood pressure variability; Q, quartile.

The Kaplan-Meier survival curves comparing patients in Q_4_ and patients in Q_1−3_ also demonstrated that a higher systolic BPV was significantly associated with higher 30-day, 90-day, and 4-year mortality (Figure 1).

### Subgroup Analyses and Sensitivity Analyses

In general, after full adjustment, patients with a higher CV of SBP were associated with a higher risk of 30-day mortality across the various subgroups (Table 3).

**Table 3:** Risk of 30-day mortality for the highest quartile (Q4) vs. Q1–3 for the coefficient of variation of SBP in different subgroups.

**Variables**	**Group**	**Number of patients**	**OR (95% CI)**	***P* for interaction**
Age	<70 years	2,325	2.38 (1.15–4.94)	0.573
	≥70 years	2,052	1.73 (0.97–3.10)	
Sex	Male	3,333	2.50 (1.44–4.34)	0.134
	Female	1,176	1.07 (0.47–2.42)	
CHF	Yes	1,224	1.44 (0.78–2.69)	0.203
	No	3,285	2.29 (1.17–4.48)	
Hypertension	Yes	3,078	1.76 (0.88–3.50)	0.998
	No	1,431	2.18 (1.18–4.02)	
AMI	Yes	371	1.29 (0.40–4.18)	0.415
	No	4,138	2.06 (1.24–3.41)	
Diabetes mellitus	Yes	1,791	1.25 (0.61–2.56)	0.076
	No	2,718	2.72 (1.47–5.03)	
Antihypertensive medication	Yes	3,383	1.78 (0.99–3.22)	0.787
	No	1,126	2.04 (0.97–4.30)	
Vasoactive medication	Yes	2,887	1.71 (1.01–2.90)	0.466
	No	1,622	3.36 (1.30–8.66)	

*ORs (95% CIs) were derived from a logistic regression model adjusted for age, sex, CHF, hypertension, AMI, diabetes mellitus, respiratory failure, peripheral vascular disease, end-stage renal disease, antihypertensive medication, vasoactive medication, and the number of blood pressure measurements*.

*AMI, acute myocardial infarction; CHF, congestive heart failure; Q, quartile; SBP, systolic blood pressure*.

In the sensitivity analyses, similar patterns can be observed when evaluating BPV as the SD of SBP, but the difference was not significant for ICU mortality or 4-year mortality (Supplementary Table 2). When we estimated BPV as the CV or SD of DBP, the results remained generally consistent, but most of the results were statistically insignificant after adjustment (Supplementary Tables 3, 4). Then, patients who were discharged or died within 12 h were excluded, and analyses based on BP records within 12 h were performed. The results revealed a similar association, with no significant difference observed for ICU, 30-day or 4-year mortality when fully adjusted (Supplementary Table 5). In addition, patients who were discharged within the first 24 h or had BP measured <10 times were also included in the analysis and the results remained consistent. Finally, we compared the survival curves of different BPV quartiles, and the difference was not significant between Q1 and Q3 for 30- and 90-day survival, while an obviously higher mortality was seen for Q4. For 4-year survival, a significant difference was observed between the four groups (Figure 1).

## Discussion

To the best of our knowledge, this is the first study with a large dataset to demonstrate an association between post-operative BPV and the prognosis of patients undergoing CABG. Higher systolic BPV was associated with a higher risk of early mortality, including ICU, 30- and 90-day mortality. However, the association between higher BPV and 4-year mortality was not significant.

As early as 2010, Aronson et al. reported that intraoperative BPV was associated with 30-day mortality in patients undergoing CABG (13). Early outcomes can be predicted from intraoperative BPV, but its clinical significance is limited, as cardiopulmonary bypass and vasoactive agents are routinely used to stabilize the BP and maintain organic perfusion when the heart is not beating. In 2019, Dyke et al. analyzed 405 patients and demonstrated that preoperative 3-year visit-to-visit BPV was a risk factor for adverse outcomes after CABG (12). However, the prognostic value of preoperative visit-to-visit BPV is restricted by the inconvenience of follow-up prior to surgery, patient non-compliance, and the uncertainty of surgical timing while post-operative short-term BPV based on ICU monitoring records is a clinically applicable prognostic factor with practical clinical utility for patients undergoing CABG.

Although various studies have reported the relationship between higher BPV and the risk of cardiovascular events, the measurement, and assessment of BPV have not been standardized, and BPV could be measured in various ways, including visit-to-visit, day-to-day and beat-to-beat variability (17). Each has its strength and limits. For healthy individuals or patients with chronic diseases such as chronic kidney disease that require long-term follow-up, visit-to-visit BPV could provide additional predictive value for long-term cardiovascular events (6, 18). For patients with acute illnesses such as intracerebral hemorrhage and patients undergoing surgical treatment, short-term post-admission or post-operative BPV is easier to acquire and more appropriate to be considered as a risk factor (19, 20). In addition, BPV is commonly evaluated based on CV and SD, while the average real variability and variation independent of the mean have also been suggested (21, 22). Regardless of which approach is selected for evaluation, methodological problems, including mortal time bias, informatics censors, inappropriate adjustment, and inconsistent equipment, can inevitably lead to potential bias (23).

In our study, systolic BPV within the first post-operative 24-h was evaluated by CV, and sensitivity analyses were performed by measuring BPV as SD quartiles of SBP and CV or SD quartiles of DBP. In addition, logistic and Cox regression analyses based on different models and subgroup analyses were conducted, and the results of the sensitivity analyses were generally consistent, though the results based on DBP were statistically insignificant. To evaluate whether post-operative 12-h BPV was associated with the prognosis, we conducted an analysis based on the post-operative 12-h CV of SBP. Although similar trends could be observed, the results were statistically insignificant when fully adjusted. One possible explanation was that within the first 12 h, patients were in unstable conditions and primarily affected by the surgical operation and intraoperative anesthesia, analgesia, and vasoactive medication usage. In addition, volume adjustment and medical titration at the beginning of ICU admission also influences the fluctuation of BP (24). As SBP is considered to be more sensitive than DBP (14, 15) and CV can better balance the difference of baseline BP compared with SD, the first 24-h systolic BPV evaluated by CV was chosen as a more rational parameter to demonstrate the association between post-operative BPV and the prognosis in patients undergoing CABG.

Compared with 4-year mortality, the association between BPV and ICU, 30- and 90-day mortality were more replicable in the analyses, indicating that the prognostic value of BPV was more prominent for short-term outcomes. For long-term survival, although the results of the Cox regression analyses demonstrated no significant difference between the two groups, the trend still favored a higher risk in the highest quartile, and the survival curves of different quartiles were separated from each other with a *P* < 0.01. The insignificance was clinically reasonable since more death attributed to other unknown confounding factors occurred as time went on, and of note is that if patients could survive the initial hemodynamic instability, they were unlikely to have long-term outcome issues.

On the basis of evidence proposed by the guidelines (25), short-term 24-h BPV is considered for risk stratification in populations or cohorts while this parameter has not come into routine usage in the current clinical practice. For better observation and treatment after the CABG operation, patients are sent to the ICU where their vital signs can be monitored and recorded in the electronic health records. The evaluation of BPV supports doctors in identifying potential patients with hemodynamic instability, which might be reflective of other issues like multi-system organ dysfunction syndrome, and making timely clinical decisions based on the oscillations of BP. Although limited therapeutic strategy targeting post-operative BPV was reported, long-acting antihypertensive drugs like amlodipine and chlorthalidone were recommended to reduce long-term BPV (26, 27). However, the optimal approaches in BPV management and whether these approaches indeed bring benefits to patients, namely, an independent reduction in adverse events and mortality, need further investigations.

The current study must be interpreted within the context of its limitation. Considering most of our included patients have received medications that limit the natural variation of BP, this would be expected to bias our results toward statistical insignificance. However, analyses based on different models and parameters consistently yielded confirmatory results. Additionally, the definition of comorbidities based on the ICD-9-CM codes would be potentially not specific enough, and certain important information including preoperative risk scores, urgent or emergent surgery, intraoperative details, and cardiovascular events were not available in the database. Finally, the nature of single-center-based cohort limits the generalizability of our conclusions and further well-designed studies should be conducted to further investigate the cause-effect relationship, prognostic, and therapeutic implication of post-operative BPV.

## Conclusions

In conclusion, our findings demonstrated that a higher systolic BPV within the first post-operative 24 h was associated with a higher risk of early mortality in patients undergoing CABG while the correlation was not significant for late mortality. Post-operative BPV can support doctors in identifying patients with potential hemodynamic instability and making timely clinical decisions.

## Data Availability Statement

The raw data supporting the conclusions of this article will be made available by the authors, without undue reservation.

## Ethics Statement

The studies involving human participants were reviewed and approved by the Massachusetts Institute of Technology (Cambridge, MA) and the Institutional Review Boards of Beth Israel Deaconess Medical Center (Boston, MA). Written informed consent for participation was not required for this study in accordance with the national legislation and the institutional requirements.

## Author Contributions

ML and XZ: conception and design. ZW and LL: administrative support. ZZ, JH, and GF: provision of study materials or patients. SC, SH, YY, LS, and KW: selection and assembly of data. ZZ, JC, and ML: data analysis and interpretation. All authors contributed to the article and approved the submitted version.

## Conflict of Interest

The authors declare that the research was conducted in the absence of any commercial or financial relationships that could be construed as a potential conflict of interest.

## Publisher's Note

All claims expressed in this article are solely those of the authors and do not necessarily represent those of their affiliated organizations, or those of the publisher, the editors and the reviewers. Any product that may be evaluated in this article, or claim that may be made by its manufacturer, is not guaranteed or endorsed by the publisher.
