# Supplementary material for: Association of Post-operative Systolic Blood Pressure Variability With Mortality After Coronary Artery Bypass Grafting
Source: Front Cardiovasc Med. 2021 Aug 12;8:717073. doi: 10.3389/fcvm.2021.717073 (PMC8387866; doi:10.3389/fcvm.2021.717073)
Supplement: Supplementary file 1 [file Data_Sheet_1.DOCX]

Table S1. Definition of comorbidities based on the International Classification of Diseases, Ninth Revision, Clinical Modification (ICD-9-CM) codes.

| Comorbidities | ICD-9-CM |
| --- | --- |
| Congestive heart failure | 428.0 |
| Hypertension | 401.0, 401.1, 401.9 |
| Acute myocardial infarction | 410.0-410.92 |
| Diabetes mellitus | 250.0-250.93 |
| Respiratory failure | 518.81, 518.83, 518.84 |
| Peripheral vascular disease | 443.81, 443.89, 443.9 |
| End stage renal disease | 285.6 |

Table S2. Association between the highest quartile (Q4) versus Q1-3 for the standardized deviation of SBP within the first 24 hours of postoperative ICU admission and mortality.

| Events | No. of events (%) | Univariable analysis | Multivariable analysis | | | |
| --- | --- | --- | --- | --- | --- | --- |
|  |  |  | Model 1 | Model 2 | Model 3 | Model 4 |
|  |  | **OR (95% CI)** | | | | |
| **ICU mortality** |  |  |  |  |  |  |
| Q1-3 | 31 (0.91) | 1.00 (Reference) | | | | |
| Q4 | 19 (1.69) | 1.85 (1.04-3.29) | 1.67 (0.93-2.99) | 1.55 (0.85-2.85) | 1.56 (0.85-2.88) | 1.50 (0.81-2.77) |
| **30-day mortality** |  |  |  |  |  |  |
| Q1-3 | 54 (1.60) | 1.00 (Reference) | | | | |
| Q4 | 34 (3.02) | 1.92 (1.24-2.96) | 1.75 (1.13-2.72) | 1.67 (1.06-2.63) | 1.68 (1.06-2.64) | 1.60 (1.01-2.53) |
|  |  | **HR (95% CI)** | | | | |
| **90-day mortality** |  |  |  |  |  |  |
| Q1-3 | 101 (2.99) | 1.00 (Reference) | | | | |
| Q4 | 59 (5.24) | 1.78 (1.29-2.45) | 1.51 (1.09-2.10) | 1.49 (1.08-2.06) | 1.50 (1.08-2.07) | 1.48 (1.07-2.06) |
| **4-year mortality** |  |  |  |  |  |  |
| Q1-3 | 306 (14.83) | 1.00 (Reference) | | | | |
| Q4 | 166 (20.02) | 1.38 (1.14-1.68) | 1.18 (0.97-1.43) | 1.15 (0.95-1.39) | 1.14 (0.95-1.39) | 1.15 (0.95-1.39) |

Model 1 was adjusted for age and sex;

Model 2 was adjusted for age, sex, CHF, hypertension, AMI, diabetes mellitus, respiratory failure, peripheral vascular disease and ESRD;

Model 3 was adjusted for the variables in Model 2 plus postoperative antihypertensive and vasoactive medication within the first 24 hours;

Model 4 was adjusted for the variables in Model 3 plus the number of blood pressure measurements within the first 24 hours.

Abbreviations: AMI, acute myocardial infarction; CHF, congestive heart failure; ESRD, end-stage renal disease; ICU, intensive care unit; Q, quartile; SBP, systolic blood pressure.

Table S3. Association between the highest quartile (Q4) versus Q1-3 for the coefficient of variation of DBP within the first 24 hours of postoperative ICU admission and mortality.

| Events | No. of events (%) | Univariable analysis | Multivariable analysis | | | |
| --- | --- | --- | --- | --- | --- | --- |
|  |  |  | Model 1 | Model 2 | Model 3 | Model 4 |
|  |  | **OR (95% CI)** | | | | |
| **ICU mortality** |  |  |  |  |  |  |
| Q1-3 | 30 (0.89) | 1.00 (Reference) | | | | |
| Q4 | 20 (1.77) | 2.02 (1.14-3.57) | 1.85 (1.04-3.30) | 1.47 (0.79-2.72) | 1.40 (0.75-2.61) | 1.37 (0.74-2.56) |
| **30-day mortality** |  |  |  |  |  |  |
| Q1-3 | 53 (1.57) | 1.00 (Reference) | | | | |
| Q4 | 35 (3.11) | 2.01 (1.31-3.10) | 1.87 (1.21-2.89) | 1.61 (1.02-2.55) | 1.55 (0.98-2.46) | 1.51 (0.95-2.39) |
|  |  | **HR (95% CI)** | | | | |
| **90-day mortality** |  |  |  |  |  |  |
| Q1-3 | 102 (3.02) | 1.00 (Reference) | | | | |
| Q4 | 58 (5.15) | 1.73 (1.25-2.39) | 1.53 (1.10-2.12) | 1.29 (0.92-1.80) | 1.23 (0.88-1.72) | 1.23 (0.88-1.72) |
| **4-year mortality** |  |  |  |  |  |  |
| Q1-3 | 333 (15.14) | 1.00 (Reference) | | | | |
| Q4 | 139 (20.09) | 1.35 (1.11-1.65) | 1.35 (1.03-1.53) | 1.22 (0.99-1.49) | 1.20 (0.98-1.47) | 1.20 (0.98-1.47) |

Model 1 was adjusted for age and sex;

Model 2 was adjusted for age, sex, CHF, hypertension, AMI, diabetes mellitus, respiratory failure, peripheral vascular disease and ESRD;

Model 3 was adjusted for the variables in Model 2 plus postoperative antihypertensive and vasoactive medication within the first 24 hours;

Model 4 was adjusted for the variables in Model 3 plus the number of blood pressure measurements within the first 24 hours.

Abbreviations: AMI, acute myocardial infarction; CHF, congestive heart failure; DBP, diastolic blood pressure; ESRD, end-stage renal disease; ICU, intensive care unit; Q, quartile.

Table S4. Association between the highest quartile (Q4) versus Q1-3 for the standardized deviation of DBP within the first 24 hours of postoperative ICU admission and mortality.

| Events | No. of events (%) | Univariable analysis | Multivariable analysis | | | |
| --- | --- | --- | --- | --- | --- | --- |
|  |  |  | Model 1 | Model 2 | Model 3 | Model 4 |
|  |  | **OR (95% CI)** | | | | |
| **ICU mortality** |  |  |  |  |  |  |
| Q1-3 | 33 (0.98) | 1.00 (Reference) | | | | |
| Q4 | 17 (1.51) | 1.55 (0.86-2.80) | 1.5 (0.83-2.72) | 1.31 (0.70-2.43) | 1.29 (0.69-2.41) | 1.27 (0.68-2.38) |
| **30-day mortality** |  |  |  |  |  |  |
| Q1-3 | 58 (1.71) | 1.00 (Reference) | | | | |
| Q4 | 30 (2.66) | 1.57 (1.00-2.45) | 1.53 (0.98-2.39) | 1.39 (0.88-2.21) | 1.37 (0.86-2.17) | 1.34 (0.84-2.14) |
|  |  | **HR (95% CI)** | | | | |
| **90-day mortality** |  |  |  |  |  |  |
| Q1-3 | 109 (3.22) | 1.00 (Reference) | | | | |
| Q4 | 51 (4.53) | 1.42 (1.02-1.98) | 1.37 (0.98-1.91) | 1.26 (0.90-1.76) | 1.23 (0.88-1.72) | 1.22 (0.87-1.71) |
| **4-year mortality** |  |  |  |  |  |  |
| Q1-3 | 356 (16.23) | 1.00 (Reference) | | | | |
| Q4 | 116 (16.62) | 1.03 (0.83-1.26) | 1.05 (0.85-1.29) | 1.02 (0.83-1.26) | 1.01 (0.81-1.25) | 1.01 (0.81-1.25) |

Model 1 was adjusted for age and sex;

Model 2 was adjusted for age, sex, CHF, hypertension, AMI, diabetes mellitus, respiratory failure, peripheral vascular disease and ESRD;

Model 3 was adjusted for the variables in Model 2 plus postoperative antihypertensive and vasoactive medication within the first 24 hours;

Model 4 was adjusted for the variables in Model 3 plus the number of blood pressure measurements within the first 24 hours.

Abbreviations: AMI, acute myocardial infarction; CHF, congestive heart failure; DBP, diastolic blood pressure; ESRD, end-stage renal disease; ICU, intensive care unit; Q, quartile.

Table S5. Association between the highest quartile (Q4) versus Q1-3 for the coefficient of variation of SBP within the first 12 hours of postoperative ICU admission and mortality.

| Events | No. of events (%) | Univariable analysis | Multivariable analysis | | | |
| --- | --- | --- | --- | --- | --- | --- |
|  |  |  | Model 1 | Model 2 | Model 3 | Model 4 |
|  |  | **OR (95% CI)** | | | | |
| **ICU mortality** |  |  |  |  |  |  |
| Q1-3 | 33 (0.94) | 1.00 (Reference) | | | | |
| Q4 | 18 (1.54) | 1.65 (0.92-2.94) | 1.51 (0.84-2.69) | 1.44 (0.79-2.63) | 1.42 (0.78-2.60) | 1.48 (0.80-2.73) |
| **30-day mortality** |  |  |  |  |  |  |
| Q1-3 | 57 (1.63) | 1.00 (Reference) | | | | |
| Q4 | 19 (2.49) | 1.54 (0.98-2.42) | 1.44 (0.91-2.27) | 1.37 (0.86-2.18) | 1.36 (0.85-2.17) | 1.38 (0.86-2.22) |
|  |  | **HR (95% CI)** | | | | |
| **90-day mortality** |  |  |  |  |  |  |
| Q1-3 | 104 (2.97) | 1.00 (Reference) | | | | |
| Q4 | 54 (4.63) | 1.57 (1.13-2.18) | 1.41 (1.02-1.97) | 1.36 (0.98-1.90) | 1.36 (0.98-1.89) | 1.40 (1.00-1.96) |
| **4-year mortality** |  |  |  |  |  |  |
| Q1-3 | 322 (14.70) | 1.00 (Reference) | | | | |
| Q4 | 149 (18.28) | 1.27 (1.05-1.54) | 1.18 (0.97-1.44) | 1.14 (0.94-1.39) | 1.14 (0.94-1.39) | 1.18 (0.97-1.44) |

Model 1 was adjusted for age and sex;

Model 2 was adjusted for age, sex, CHF, hypertension, AMI, diabetes mellitus, respiratory failure, peripheral vascular disease and ESRD;

Model 3 was adjusted for the variables in Model 2 plus postoperative antihypertensive and vasoactive medication within the first 12 hours;

Model 4 was adjusted for the variables in Model 3 plus the number of blood pressure measurements within the first 12 hours.

Abbreviations: AMI, acute myocardial infarction; CHF, congestive heart failure; ESRD, end-stage renal disease; ICU, intensive care unit; Q, quartile; SBP, systolic blood pressure.

Table S6. Association between the highest quartile (Q4) versus Q1-3 for the coefficient of variation of SBP within the first 24 hours of postoperative ICU admission and mortality, after included patients discharged within 24 hours or had BP measured less than ten times.

| Events | No. of events (%) | Univariable analysis | Multivariable analysis | | | |
| --- | --- | --- | --- | --- | --- | --- |
|  |  |  | Model 1 | Model 2 | Model 3 | Model 4 |
|  |  | **OR (95% CI)** | | | | |
| **ICU mortality** |  |  |  |  |  |  |
| Q1-3 | 31 (0.87) | 1.00 (Reference) | | | | |
| Q4 | 26 (2.20) | 2.55 (1.51-4.32) | 2.30 (1.35-3.90) | 2.15 (1.24-3.72) | 2.05 (1.17-3.56) | 2.05 (1.17-3.59) |
| **30-day mortality** |  |  |  |  |  |  |
| Q1-3 | 53 (1.49) | 1.00 (Reference) | | | | |
| Q4 | 42 (3.55) | 2.43 (1.61-3.66) | 2.23 (1.47-3.37) | 2.09 (1.36-3.20) | 1.99 (1.29-3.06) | 1.93 (1.25-2.99) |
|  |  | **HR (95% CI)** | | | | |
| **90-day mortality** |  |  |  |  |  |  |
| Q1-3 | 100 (2.82) | 1.00 (Reference) | | | | |
| Q4 | 68 (5.75) | 2.08 (1.53-2.83) | 1.83 (1.34-2.50) | 1.74 (1.28-2.37) | 1.68 (1.23-2.30) | 1.70 (1.24-2.33) |
| **4-year mortality** |  |  |  |  |  |  |
| Q1-3 | 322 (14.61) | 1.00 (Reference) | | | | |
| Q4 | 166 (19.37) | 1.37 (1.14-1.65) | 1.23 (1.02-1.50) | 1.19 (0.98-1.43) | 1.19 (0.98-1.43) | 1.20 (0.99-1.45) |

Model 1 was adjusted for age and sex;

Model 2 was adjusted for age, sex, CHF, hypertension, AMI, diabetes mellitus, respiratory failure, peripheral vascular disease and ESRD;

Model 3 was adjusted for the variables in Model 2 plus postoperative antihypertensive and vasoactive medication within the first 12 hours;

Model 4 was adjusted for the variables in Model 3 plus the number of blood pressure measurements within the first 12 hours.

Abbreviations: AMI, acute myocardial infarction; CHF, congestive heart failure; ESRD, end-stage renal disease; ICU, intensive care unit; Q, quartile; SBP, systolic blood pressure.
